# Supplementary material for: Identification of photoperiod-induced specific miRNAs in the adrenal glands of Sunite sheep (Ovis aries)
Source: Front Vet Sci. 2022 Jul 22;9:888207. doi: 10.3389/fvets.2022.888207 (PMC9354845; doi:10.3389/fvets.2022.888207)
Supplement: Supplementary file 1 [file Data_Sheet_1.ZIP › Supplementary file/miRDeep2 file.zip!/miRDeep2 file/Novel_Structure/LP42A2/Novel_501.pdf]

Provisional ID	:	NC_019467.2_19352
Score total	:	1.4
Score for star read(s)	:	-1.3
Score for read counts	:	0
Score for mfe	:	1.1
Score for randfold	:	1.6
Score for cons. seed	:	
Total read count	:	22
Mature read count	:	22
Loop read count	:	0
Star read count	:	0

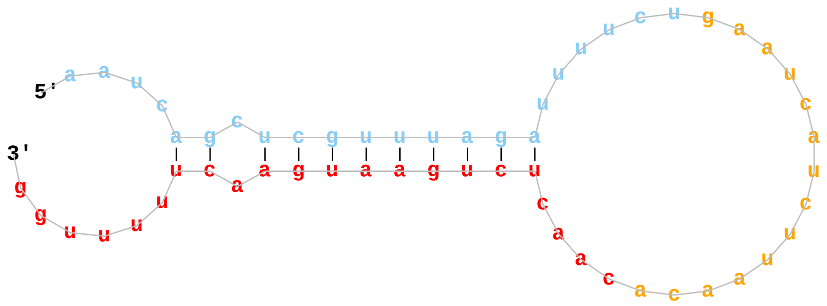

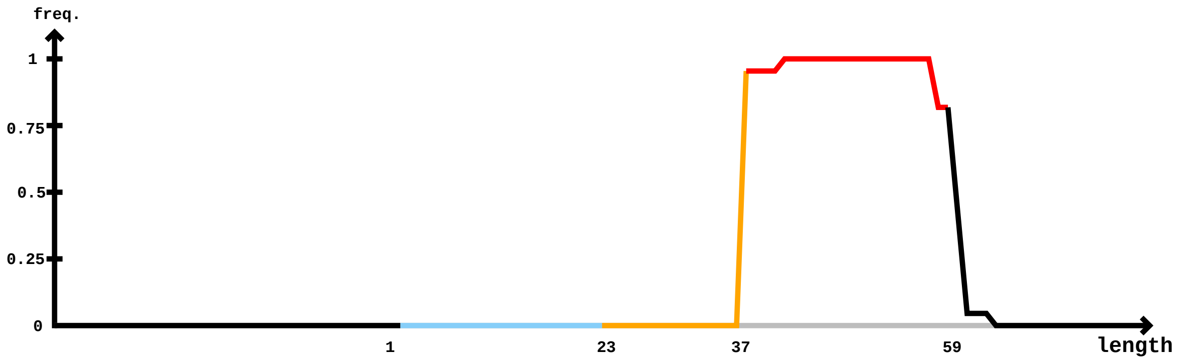

Star **Mature**

```
5'- uucugagcuugguauuagugauauuggcuuggcuaaucagcucguuuagauuucugaaucaucuuaacacaacucugaaugaacuuuuuggccagcccaaauacaagcuca   -3'    exp
    ...((((((((.((((..((.....((((.((((((..((.(((((((((.......................))))))))).))..))))))))))))))))))))))))    reads    mm    sample
    .....................................................................caacucugaaugaacuuuuu......................    4        0     seq
    .....................................................................caacucugaaugaacuuuuugg....................    6        0     seq
    .....................................................................Aaacucugaaugaacuuuuugg....................    11       1     seq
    .........................................................................Ccugaaugaacuuuuuggccag................    1        1     seq
```
